# Personalized Medicine in Cerebrovascular Neurosurgery: Precision Neurosurgical Management of Cerebral Aneurysms and Subarachnoid Hemorrhage

**DOI:** 10.3389/fsurg.2016.00034

**Published:** 2016-06-22

**Authors:** Achal Singh Achrol, Gary K. Steinberg

**Affiliations:** ^1^Department of Neurosurgery, Stanford University School of Medicine, Stanford, CA, USA

**Keywords:** biomarkers, cerebral aneurysm, neuroimaging, radiogenomics, subarachnoid hemorrhage

## Abstract

Cerebral aneurysms are common vascular lesions. Little is known about the pathogenesis of these lesions and the process by which they destabilize and progress to rupture. Treatment decisions are motivated by a desire to prevent rupture and the devastating morbidity and mortality associated with resulting subarachnoid hemorrhage (SAH). For patients presenting with SAH, urgent intervention is required to stabilize the lesion and prevent re-rupture. Those patients fortunate enough to survive a presenting SAH and subsequent securing of their aneurysm must still face a spectrum of secondary sequelae, which can include cerebral vasospasm, delayed ischemia, seizures, cerebral edema, hydrocephalus, and endocrinologic and catecholamine-induced systemic dysfunction in cardiac, pulmonary, and renal systems. Increased focus on understanding the pathophysiology and molecular characteristics of these secondary processes will enable the development of targeted therapeutics and novel diagnostics for improved patient selection in personalized medicine trials for SAH. In unruptured cerebral aneurysms, treatment decisions are less clear and currently based solely on treating larger lesions, using rigid aneurysm size cutoffs generalized from recent studies that are the subject of ongoing controversy. Further compounding this controversy is the fact that the vast majority of aneurysms that come to clinical attention at the time of a hemorrhagic presentation are of smaller size, suggesting that small aneurysms are indeed not benign lesions. As such, patient-specific biomarkers that better predict which aneurysms represent high-risk lesions that warrant clinical intervention are of vital importance. Recent advancements in genomic and proteomic technologies have enabled the identification of molecular characteristics that may prove useful in tracking aneurysm growth and progression and identifying targets for prophylactic therapeutic interventions. Novel quantitative neuroimaging technologies have also recently emerged, capable of non-invasive characterization of hemodynamic factors, inflammation, and structural changes in aneurysmal walls. The combined use of these quantitative neuroimaging and molecular-based approaches, called *Radiogenomics*, is a technique that holds great promise in better characterizing individual aneurysms. In the near future, these radiogenomic techniques may help improve quality of life and patient outcomes *via* patient-specific approaches to the treatment of unruptured cerebral aneurysms and personalized medical management of secondary processes following aneurysmal SAH.

## Introduction

Cerebral aneurysms are common vascular lesions with prevalence in autopsy studies as high as 5% (1). The most common clinical presentation of cerebral aneurysms is rupture leading to subarachnoid hemorrhage (SAH) (2). The estimated incidence of SAH from ruptured intracranial aneurysms in the United States is one case per 10,000 persons (2, 3). An estimated 10% of these patients die before reaching medical attention with the 30-day mortality rate reaching as high as 45%. The 30% of patients who do survive suffer significant disability (3–5).

Aneurysms that present with SAH represent unstable lesions with significant risk of re-rupture, with recurrent hemorrhage within the first 24 h in as many as 4%, and in as many as 20% within the first 2 weeks of the initial event, if left unsecure (2). Symptomatic unruptured aneurysms presenting with new cranial nerve palsies or brainstem dysfunction are at increased risk of rupture, as high as 6% per year, and should be treated (6).

Recent advances in genomic and proteomic technologies have enabled the identification of molecular characteristics that may prove useful in tracking aneurysm growth and progression to guide treatment of unruptured aneurysms. Novel quantitative neuroimaging technologies have also recently emerged, capable of non-invasive characterization of hemodynamic factors, inflammation, and structural changes in aneurysmal walls. The combined use of these quantitative neuroimaging and molecular-based approaches, called *Radiogenomics*, is a technique that holds great promise in better characterizing individual aneurysms.

Beyond securing the aneurysm from risk of rupture, the treatment of patients with aneurysmal SAH includes managing a significant spectrum of secondary sequelae, which can include cerebral vasospasm (CV), delayed ischemia, seizures, cerebral edema, hydrocephalus, and endocrinologic and catecholamine-induced systemic dysfunction in cardiac, pulmonary, and renal systems. Optimizing management of these complex multisystem factors is critical for improving the 30-day mortality rate (as high as 45%) and the proportion of significantly disabled survivors (as high as 30%). An increased focus on understanding the pathophysiology and molecular characteristics of these secondary processes will enable the development of targeted therapeutics and novel diagnostics for improved patient selection in personalized medicine trials for SAH.

## Current Controversies in the Management of Cerebral Aneurysms

The management of asymptomatic unruptured aneurysms is the subject of ongoing controversy. A recent prospective observational cohort study, The International Study of Unruptured Intracranial Aneurysms (ISUIA), in which 1,692 patients were preselected to be conservatively followed, reported that the subgroup with the smallest aneurysms (defined in this study as <7 mm) had an observed 5-year rupture rate of 0% during the interval they were followed (1). Controversy surrounding the methodology of this study exists because, unlike a true natural history study, patients may have been preselected for inclusion on the basis of their surgeons’ opinions that these aneurysms were less likely to rupture. Consistent with this, the rupture rates of this observational cohort were significantly lower than in other studies of unruptured cerebral aneurysms (2, 7–10). Another controversy was the ISUIA-reported risk of morbidity associated with microsurgical clipping of unruptured aneurysms as 15.7% after 1 year, which raised significant concerns when compared to the literature reporting surgical morbidity in the range of 3–7% (2). The result of inappropriately generalizing the ISUIA data of a preselected subset of aneurysms has nonetheless had the important effect of at least temporarily discouraging the treatment of many unruptured cerebral aneurysms. The result this will have on actual patient outcomes in real-world populations remains to be seen. In the interim, it is vitally important to generate improved biomarkers that move past arbitrary size cutoffs so that clinicians can better characterize rupture risk in individual lesions and thus improve decision-making for each unique patient.

Moving beyond the question of when to intervene, the issue of how to intervene is also the subject of much controversy, with options including microsurgical clipping and endovascular coiling. The International Subarachnoid Aneurysm Trial (ISAT) reported prospectively randomizing 2,143 patients, who presented with ruptured aneurysms, to either clipping or coiling (11). an important caveat of this analysis is that these 2,143 patients represented only a fraction of the total 9,559 patients the study initially assessed with aneurysmal SAH. The vast majority of real-world aneurysmal SAH patients (77.6%) were excluded upfront from this analysis, based on inclusion criteria that resulted in an analysis of a minority of aneurysmal SAH patients. The clinical characteristics of the resulting study demonstrated the profound effects of this selection bias, including 90% having favorable clinical grade, 95% having aneurysms in the anterior circulation, and 90% of aneurysms being <10 mm. Generalizing these findings may be inappropriate, and in fact many contributors to the ISAT trial have themselves pointed out significant issues with data transparency and need for secondary sources of data on this critical topic (12). As a result of these significant limitations of ISUIA and ISAT, and despite the impact they have already had on current treatments, whether to observe, surgically treat, or endovascularly manage intracranial aneurysms remains controversial.

Whether the increased durability of clipping outweighs its slightly higher risks compared to coiling is unknown. In fact, even ISAT investigators reported that the rehemorrhage rates and recoiling rates in subsequent analyses of their data indicate significant problems with the study’s original conclusions (12). Nevertheless, endovascular technology is likely to continue to advance with indications and outcomes likely to be constantly changing.

As such, patient-specific biomarkers that better predict which aneurysms represent high-risk lesions and which lesions are likely to respond best to a particular therapy are of vital importance.

## Emerging Biomarkers in the Management of Unruptured Cerebral Aneurysms

Although the pathogenesis of cerebral aneurysms is unknown, their development at stereotyped locations associated with specific hemodynamic factors suggests that regional blood flow patterns play a fundamental role in the pathophysiology of the disease (13–16), as recently reviewed by Can and Du (17). Using non-invasive quantitative imaging to characterize aneurysm morphology and computational fluid dynamics analyses of resulting hemodynamics, these studies have provided new insight into the key factors that play in a role in aneurysm progression and risk of rupture. Interestingly, bifurcation aneurysms were associated with high wall shear stress (WSS), suggesting that wall remodeling and degeneration *via* endothelial injury is of greatest relevance in these aneurysms. In contrast, sidewall aneurysms were associated with low WSS, suggesting that stasis of blood flow, and resulting endothelial dysfunction with pro-inflammatory-mediated degeneration of the aneurysm wall, may be more clinically relevant in these aneurysms (17). In paired analysis of unruptured aneurysms that went on to rupture, the hemodynamic factors associated with rupture risk included low shear index area (LSA), defined as the area of the aneurysm wall exposed to a WSS <10% of the mean parent vessel, which was observed to be higher in aneurysms that went on to rupture (i.e., a greater percentage of the aneurysm wall was exposed to low shear stresses). However, patients with ruptured aneurysms experienced a higher maximum WSS (17). Taken together, these data suggest that a significant area of low shear stress results in endothelial dysfunction and degeneration of the aneurysmal wall to the point of susceptibility, and that focally high WSS exerted against this background results in the subsequent rupture event. These hemodynamic parameters of LSA and WSS provide a more dynamic measure of the aneurysm than arbitrary size measurement cutoffs proposed by the ISUIA and ISAT studies, and these next generation parameters will likely play an increasing role in the patient-specific characterization of aneurysms and associated clinical decision-making in the future.

Recently, ferumoxytol-enhanced magnetic resonance imaging has shown promise in non-invasively characterizing aneurysm inflammation. Increased ferumoxytol uptake in aneurysm walls is a measure of myeloid cell inflammation, and has predicted aneurysm instability and an increased 6-month rupture risk in pilot studies. Thus, increased ferumoxytol uptake may serve as a biomarker for lesions warranting urgent intervention (18–20). As hemodynamic factors, such as high LSA, may result in a pro-inflammatory milieu, with subsequent endothelial apoptosis and aneurysmal wall degeneration (17), a combination of hemodynamic and inflammatory characterization by newer non-invasive neuroimaging modalities may become increasingly important in the patient-specific management of aneurysms in the near future.

## Emerging Biomarkers in the Management of Secondary Sequelae of SAH

Secondary sequelae of SAH include CV, delayed ischemia, seizures, cerebral edema, hydrocephalus, and endocrinologic and catecholamine-induced systemic dysfunction in cardiac, pulmonary, and renal systems. Currently there are no established biomarkers for preclinical diagnosis or monitoring of progression of these secondary sequelae.

Hydrocephalus can develop in up to 20% of patients who have aneurysmal SAH (2), requiring ventriculostomy for drainage of cerebrospinal fluid (CSF). There are currently no accurate predictors of shunt dependency after ventriculostomy placement in SAH, but emerging CSF-based biomarkers that reflect the rate of CSF clearance, as well as neuroimaging that quantifies CSF dynamics, hold promise in selecting patients for rapid removal of the external ventricular drain to minimize risks of ventriculitis.

Cerebral vasospasm is a major cause of morbidity and mortality in SAH and refers to intracranial vasoconstriction that may occur between 3 and 14 days after SAH. The pathogenesis of vasospasm is unknown and even with maximal therapy vasospasm can cause strokes and death (21). Approximately two-thirds of all patients with SAH who undergo cerebral angiography will demonstrate radiographic evidence of vasospasm, known as angiographic CV. Symptomatic (clinical) CV, defined as the development of new focal neurologic deficits in patients with SAH in association with angiographic CV and not attributable to other causes, occurs in approximately one-third of all patients with SAH. Approximately one-third of these patients with CV die from the CV-related infarcts and another one-third are left significantly disabled. Medical treatment of CV consists of orally administered nimodipine (60 mg every 4 h for 21 days), which has been shown to improve outcome after SAH (22). Patients are monitored with daily transcranial Doppler (TCD) velocities, and in patients who develop elevated TCDs and new neurologic deficits, triple-H therapy is initiated (hypertension, hypervolemia, and hemodilution) (3). Patients with persisting neurologic deficit undergo urgent catheter angiography to confirm the presence of vasospasm and if confirmed are treated with intra-arterial administration of smooth muscle relaxants, such as papaverine or nicardipine or with balloon angioplasty. These antispasmodic therapies can result in angiographically confirmed arterial dilatation in >90% of patients (23–25).

Multiple CSF biomarkers have been identified for the early diagnosis of symptomatic CV, as recently reviewed by Lad et al. (26), which may help guide patient-specific selection for personalized medicine trials aimed at preventing delayed ischemic neurologic deficits, such as protocols using earlier angiography for early intra-arterial smooth muscle relaxant therapy. Endothelin-1 has been shown to significantly increase days 4–7 after SAH in patients who develop symptomatic CV versus those who do not (27), and this increase predicts the occurrence of symptomatic CV (28). CSF interleukin (IL)-6 levels also significantly increase in the first 4–5 days after disease onset in patients with CV compared to those with uncomplicated SAH (29). These data suggest that endothelin-1 and IL-6 could be useful diagnostic and predictive markers for CV and potentially useful tools for personalized medicine protocols in the treatment and prevention of symptomatic CV.

Subarachnoid hemorrhage can result in overactivity of the sympathetic nervous system and catecholamine surge with resulting multisystem dysfunction. Cardiac abnormalities after SAH are common, including electrocardiographic changes, elevations in cardiac enzymes, and left ventricular dysfunction in up to one-third of cases (30–32). These abnormalities appear to directly result from the excessive catecholamine release in response to the intracranial hemorrhage (33). In some patients, other adverse events from this catecholamine surge include pulmonary edema, hypotension requiring vasopressors, delayed strokes, and death (34). The combination of decreased cardiac contractility, increased pulmonary vascular permeability, increased pulmonary vascular pressure, and increased volume from resuscitation results in the development of this pulmonary edema, and increased preload results in stretching of the cardiac atrium and atrial natriuretic peptide release (peaks on day 2) (35). This natriuretic peptide acts on renal tubules, triggering sodium and volume loss, and without appropriate resuscitation, plasma sodium levels fall significantly by post-rupture days 4–6, which can be preempted by judicious volume and salt replacement. This has been shown to reduce the incidence of severe CV (36). The relationship between natriuretic and diuretic states after aneurysmal SAH and the subsequent development of CV, particularly with regards to activation of the renin–angiotensin–aldosterone system between days 4 and 6, warrant further study and may provide further biomarkers to guide patient-specific treatments that optimize sodium and fluid balance, address natriuretic and renin–angiotensin–aldosterone signaling dysfunction, and provide appropriate inotropic and vasopressor support during myocardial dysfunction and ventilator support during neurogenic pulmonary edema.

## Conclusion

Patient-specific biomarkers that better predict which cerebral aneurysms represent high-risk lesions worthy of intervention are of vital importance. Personalized treatment strategies are also increasingly important in the management of secondary sequelae from SAH, including CV, delayed ischemia, seizures, cerebral edema, hydrocephalus, and endocrinologic and catecholamine-induced systemic dysfunction in cardiac, pulmonary, and renal systems. The combined use of these quantitative neuroimaging and molecular-based approaches, called *Radiogenomics*, is a technique that holds great promise in better characterizing individual aneurysms. In the near future, these radiogenomic techniques may help improve quality of life and patient outcomes *via* patient-specific approaches to the treatment of unruptured cerebral aneurysms and personalized medical management of secondary processes following aneurysmal SAH.

## Author Contributions

All authors listed, have made substantial, direct and intellectual contribution to the work, and approved it for publication.

## Conflict of Interest Statement

Dr. Steinberg is a consultant for Qool Therapeutics and for Peter Lazic US, Inc. The authors declare that the research was conducted in the absence of any commercial or financial relationships that could be construed as a potential conflict of interest.
